# Atypical biological features of a new cold seep site on the Lofoten-Vesterålen continental margin (northern Norway)

**DOI:** 10.1038/s41598-018-38070-9

**Published:** 2019-02-11

**Authors:** Arunima Sen, Tobias Himmler, Wei Li Hong, Cheshtaa Chitkara, Raymond W. Lee, Benedicte Ferré, Aivo Lepland, Jochen Knies

**Affiliations:** 1 Centre for Arctic Gas Hydrate, Environment and Climate (CAGE), Department of Geosciences, UiT-The Arctic University of Norway in Tromsø, Tromsø, Norway; 2 Geological Survey of Norway (NGU), Trondheim, Norway; 3 Faculty of Science and Technology, University of Basque Country, Leioa-Bilbao, Spain; 4 School of Biological Sciences, Washington State University, Pullman, WA USA

## Abstract

A newly discovered cold seep from the Lofoten-Vesterålen margin (Norwegian Sea) is dominated by the chemosymbiotrophic siboglinid *Oligobrachia haakonmosbiensis* like other high latitude seeps, but additionally displays uncharacteristic features. Sulphidic bottom water likely prevents colonization by cnidarians and sponges, resulting in fewer taxa than deeper seeps in the region, representing a deviation from depth-related trends seen among seeps elsewhere. *O. haakonmosbiensis* was present among carbonate and barite crusts, constituting the first record of frenulates among hard substrates. The presence of both adults and egg cases indicate that *Ambylraja hyperborea* skates use the site as an egg case nursery ground. Due to sub-zero ambient temperatures (−0.7 °C), we hypothesize that small, seepage related heat anomalies aid egg incubation and prevent embryo mortality. We place our results within the context of high–latitude seeps and suggest they exert evolutionary pressure on benthic species, thereby selecting for elevated exploitation and occupancy of high-productivity habitats.

## Introduction

The breakdown and transformation of buried organic matter generates sub-seafloor hydrocarbons, including methane^1^. As methane seeps out from these reservoirs and migrates through the sediment, it is oxidized anaerobically through microbial activity, which, in concert with sulphate reduction produces hydrogen sulphide (anaerobic oxidation of methane or, AOM: CH_4_ + SO_4_^2−^ → HCO_3_^−^ + HS^−^ + H_2_O)^2^. This process creates reduced sediment conditions on the seafloor, known as cold seeps^2–5^. The reduced gases at seeps are toxic to most higher organisms because of their ability to hinder oxidative respiration^6–9^. Yet, the reducing conditions are favourable for chemosynthesis-based organisms, that utilize reduced chemical compounds for carbon fixation and autotrophy^3–5^. Consequently, seeps often host dense, biomass-rich communities, with chemosynthesis-based organisms at the base of the food chain. On the deep seafloor, far from light-dependent photosynthetic primary production, this oasis effect can be particularly manifest such that seeps tend to stand out in stark contrast to the seemingly barren, desert-like conditions of the surrounding seafloor^3,10^. Seeps also leave enduring legacies in the form of authigenic carbonates or sulphates that precipitate and form crusts during local biogeochemical carbon and sulphur cycling processes^11–13^. These crusts provide settlement surfaces for hard-bottom animals, including the eventual establishment of large deep-water reef systems^14,15^. Therefore, seeps can have wide-ranging impacts on the larger marine ecosystem over considerable expanses of both space and time^3^. For this reason, understanding local seep processes and subsequent impacts is critical, particularly if environmentally sound management policies are at stake.

**Figure 1:**
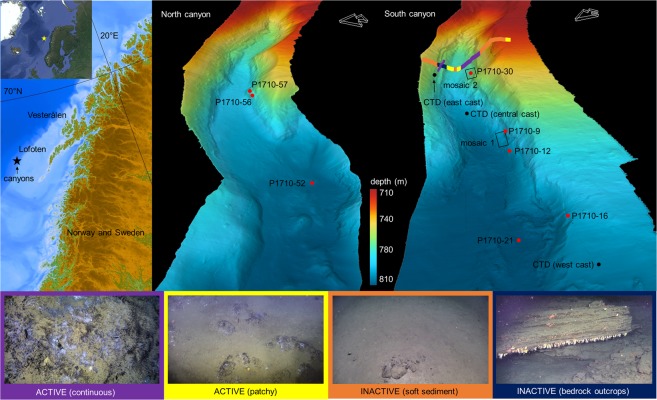
The study site. The inset on the top left shows the overall location in relation to continental Norway, and below, the Lofoten-Vesterålen region is highlighted. Satellite imagery for the inset was obtained using Google Earth Pro version 7.3 (August 22, 2018, Data SIO, NOAA, U.S. Navy, NGA, GEBCO Image Landsat/Copernicus, IBCAO, U.S. Geological Survey, http://www.earth.google.com). Bathymetry for the Lofoten Vesterålen region was obtained from IBCAO version 3.0^74^. Bathymetry maps of the two canyons are shown, with the locations of the two mosaics, CTD casts and push core samples. The multicoloured line across the south canyon shows the distribution of inactive and actively seeping areas within the canyon based on two transects. Purple represents active seepage in soft sediment where extensive chemosynthesis based communities were seen, with occasional crusts. Yellow represents active areas where chemosynthesis based communities were patchily distributed, with occasional crusts. Orange represents inactive sites with soft sediment and occasional drop stones. Dark blue represents inactive bedrock outcrops. Pictures showing these four categories are on the bottom.

In the Lofoten–Vesterålen region off northern Norway (Fig. 1), effective management policy is crucial because of the area’s ecological and economic importance^16^. It is a focal point for the interaction of warm Atlantic water with cold Arctic water, resulting in high biological productivity, including some of the largest commercial fish spawning stocks in the world^17,18^. Seabird colonies, marine mammals and coral reefs are also extensive, and the region is home to the largest *Lophelia pertusa* coldwater coral reef in the world. The sum effect is a thriving and highly profitable commercial sector, and expansion to include exploitation of the region’s vast oil and gas reserves has become a debate of national socio-economic importance^18–21^. Monitoring and survey programmes have been launched to increase knowledge and even identify key gaps in the understanding of the Lofoten–Vesterålen ecosystem, in order to update management policies and guidelines^22,23^. During this process, a previously unknown cold seep site was discovered on the lower continental slope off Lofoten–Vesterålen (68°9′ N, 10°28′ E, ~750 m water depth, Fig. 1)^24^. This discovery has novel implications for local ecosystem functioning and environmental policy because seeps can have significant ecological impacts, and are likely to be fairly widespread in this area since their occurrences often coincide with that of oil and gas reservoirs. Studying seeps is therefore a necessary endeavour if a holistic understanding of the larger marine ecosystem is desired.

In this study, we characterize this new Lofoten–Vesterålen seep site by using a multifaceted approach. We combine high-resolution imagery within a Geographic Information System (GIS), porewater geochemical profiles, CTD data, stable isotope measurements, and seep crust characterization in order to understand key features of both the biotic and abiotic aspects of the system. We highlight potential impacts and relationships with the background, non-seep benthos. Additionally, the site displays certain deviations from known characteristics of cold seeps, which has implications for cold seep biology, particularly within the context of high latitude or polar regions of the world.

## Results

The canyon site was visited in August 2017, with the research vessel *G. O. Sars*, (University of Bergen) and in July 2018 with *Helmer Hanssen* (Arctic University of Norway in Tromsø). Most of the work conducted for the purpose of this study was conducted in 2017, with the remotely operated vehicle (ROV), Ægir 6000. Both canyons were surveyed initially to gain a general, visual perspective after which detailed topographical mapping was conducted via a multibeam echosounder (Fig. 1). Porewater geochemistry was characterized via push core collections; three were taken within the northern canyon and five were taken within the southern canyon (Fig. 1). Microbial mats, siboglinid worm tufts and one location where active seepage was not visible were targeted for push core sampling. Multiple crusts were collected from both canyons and mineralogical analyses were carried out on them to understand their composition. Detailed imaging and mosaicking was undertaken to determine community composition in areas of active seepage, however, due to time constraints, this could only be conducted at two locations within the southern canyon. In 2018, three CTD casts were taken in the southern canyon in order to characterize the water column.

### The study site and areas of active or inactive seepage

**Figure 2:**
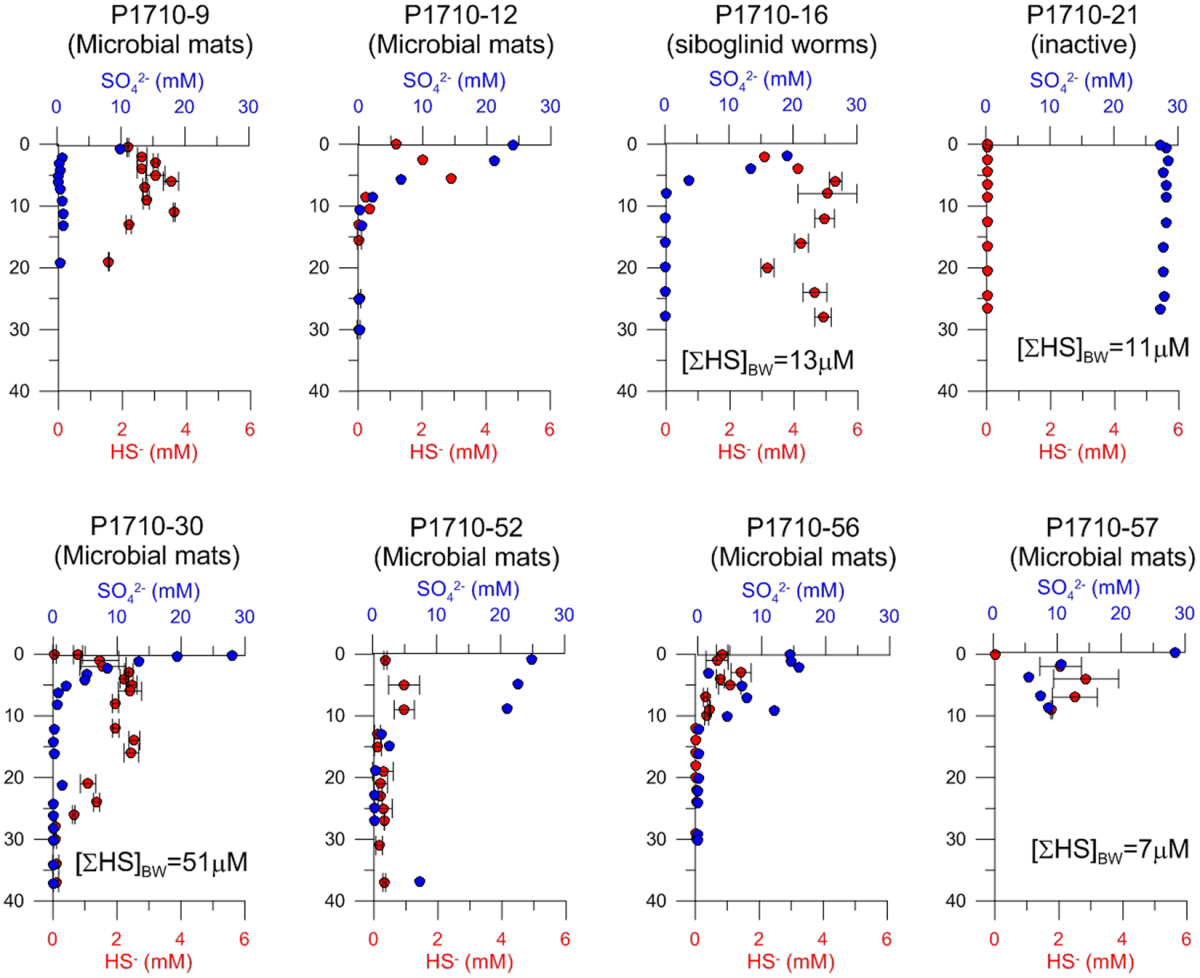
Sediment sulphide and sulphate profiles measured from push core samples taken within the two canyons. Core P1710-09 was taken just off mosaic 1 and core P1710-30 was taken in the center of mosaic 2 (see Fig. 1 for locations).

The study site consists of two adjacent canyons, north of the Trænadjupet slide on the southern part of the continental Lofoten-Vesterålen slope (Fig. 1). Both canyons are about 2 km long and ~50 m deep; water depth at their bases is about 750 m. The seafloor of both canyons comprises areas that can visually be characterized as active seepage or inactive areas. Areas hosting chemosynthesis-based organisms such as microbial mats and siboglinid annelids (*Oligobrachia haakonmosbiensis*)^25^ represent sites of active seepage. Porewater geochemical analyses revealed elevated concentrations of hydrogen sulphide (millimolar levels) in cores taken within bacterial mats and worm tufts, though the zone of sulphide availability was generally limited to about 10–20 cm below the sediment surface (Fig. 2). Sulphide was also measurable in bottom water samples (7–51 µM) and at the sediment-water interface (0.78–1.18 mM) from these locations as well (Fig. 2). No rising gas bubbles were observed during the course of our exploration of the site. Therefore, seepage appears to be limited to the dissolved phase, at least for the summer season and during our period of investigation.

Areas devoid of chemosynthesis-based organisms are referred to as inactive, non-seep areas. These areas were colonized sparsely by background benthic species in soft sediment locations, and more densely by background hard-bottom animals on bedrock outcrops (Fig. 1). Sulphide was present in small, micromolar concentrations (11–13 µM) along the length of the one core taken from an inactive, soft sediment area (P1710-21), and sulphide was also detectable in the bottom water sample from this core (11 µM, Fig. 2). Therefore, at least in soft sediment locations, the inactive areas are not representative of background conditions and are exposed to minute amounts of seepage.

It appeared that active areas occurred predominantly along the canyon flanks while inactive areas were visible at the canyon base and shoulders. Our conclusions are based on seafloor inspections along two survey transects that crossed the entire width of the southern canyon (Fig. 1). However, it should be kept in mind that this is not a thorough assessment of where active and inactive areas are located in both canyon sites. The latter requires large scale imaging to gain a more comprehensive map of the two canyons with respect to sites of active and inactive seepage.

### Chemosynthesis-based community characteristics

**Figure 3:**
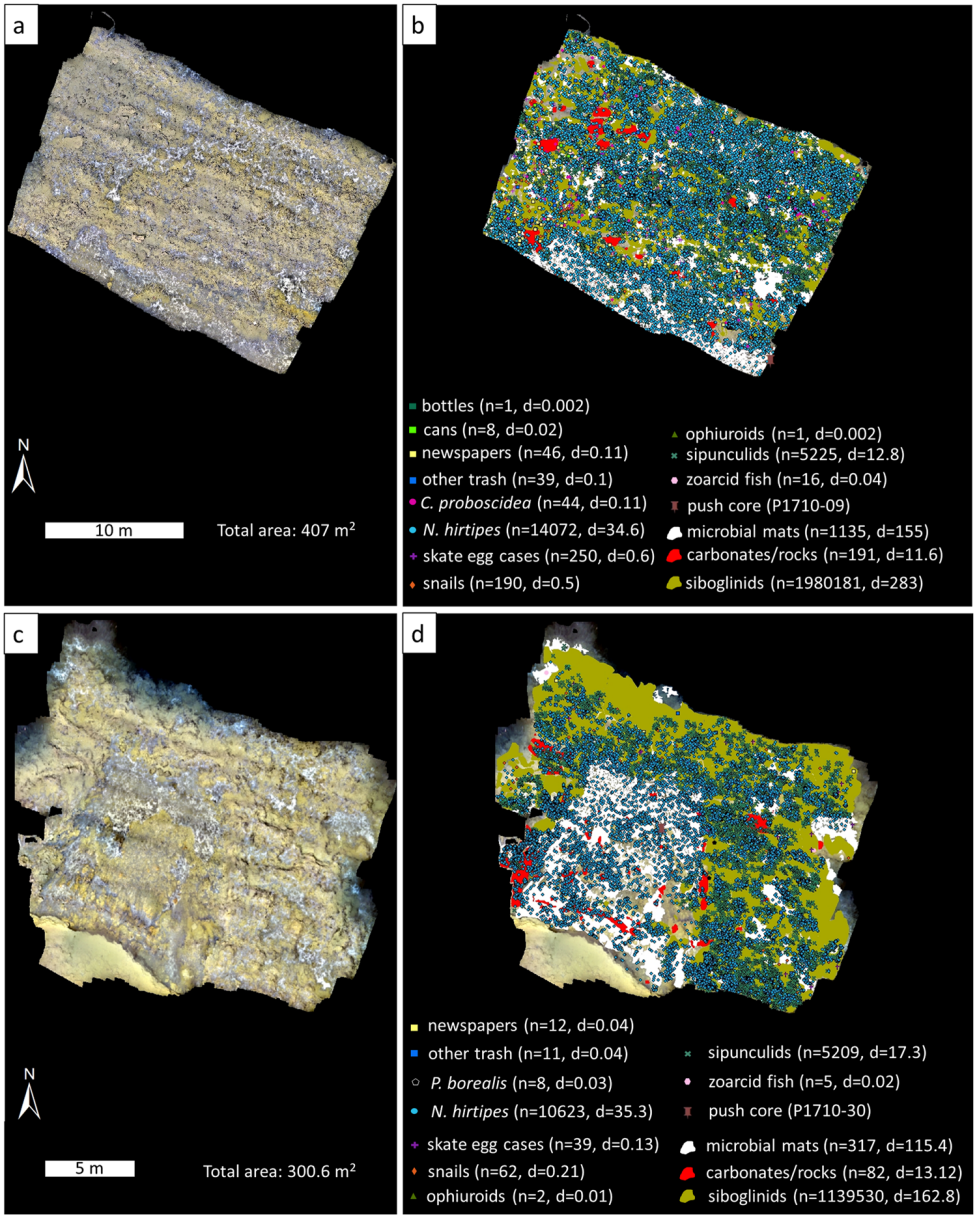
The two geroreferenced mosaics. (**a,b**) mosaic 1, (**c,d**) mosaic 2. On the left is the georeferenced mosaic and on the right is the same mosaic with all the animals and other features digitized. The numbers of individuals or polygons/aggregations (n) and density per m^2^ of the same (**d**) are listed.

**Figure 4:**
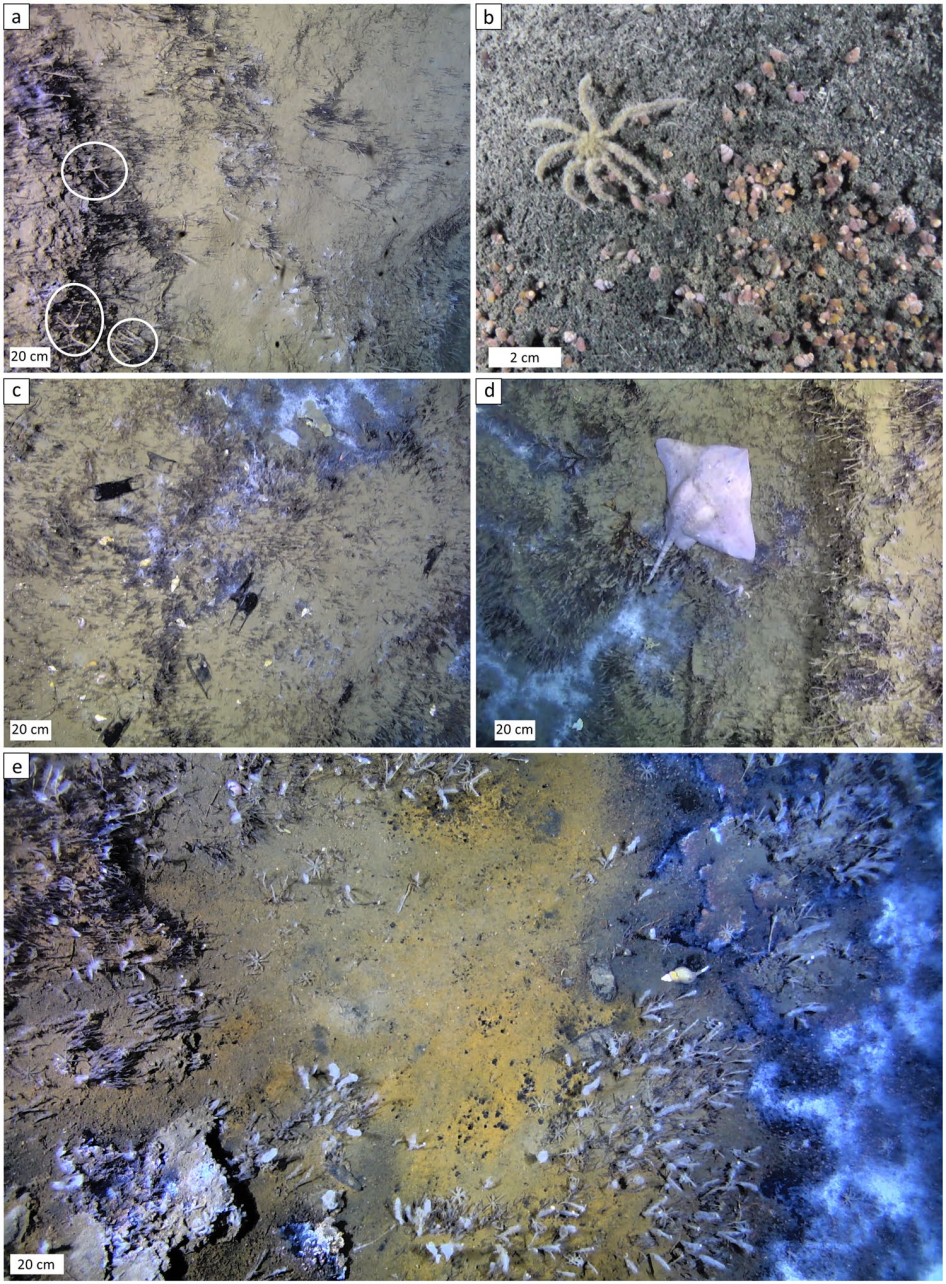
Examples of important community members and skate egg cases. (**a**) The unidentified branched animal and *O. haakonmosbiensis* aggregations. Black, hair like organisms abundant in the image are tubes of *O. haakonmosbiensis*. On many occasions, another animal, represented by slender, whitish, branched organisms were present among them (a few individuals are highlighted with circles). (**b**) Close up view of the rissoid snails that were abundant among microbial mats but could not be visualized sufficiently within the mosaics to be digitized. An *N. hirtipes* sea spider is also visible in this image. (**c**) Examples of the black, horned skate egg cases seen at the study site. Note how some are partially buried in the sediment. (**d**) An adult Arctic skate (*Ambylraja hyperborea*). (**e**) Fuzzy individuals of the unknown branched animal and *O. haakonmosbiensis* likely caused by the presence of bacterial colonies.

The fauna in active areas is characterized by low taxonomic richness. Only eight living animal taxa were seen and marked in the two georeferenced seafloor mosaics: *Oligobrachia haakonmosbiensis* frenulates, *Colossendeis proboscidea* sea spiders, *Nymphon hirtipes* sea spiders, *Pandalus borealis* shrimp, sipunculids, snails (possibly buccinids), zoarcid fish and ophiuroids (only three individuals, Fig. 3). Additionally, a branched animal (hydroid, sponge or bryozoan) was present among siboglinids, and rissoid snails occurred among microbial mats. Both of these taxa were highly abundant, even if they could not be specifically quantified (Fig. 4A,B, Supplementary Movie).

Nearest neighbour analyses suggest that the distributions of *N. hirtipes*, sipunculids and snails are clustered within the mosaicked areas (Table 1). For *N. hirtipes*, this clustering could be linked to a certain extent with microbial mats, where this species was slightly overrepresented (38% of both mosaics consisted of microbial mats and 44% and 48% of *N. hirtipes* individuals were seen among them in mosaics 1 and 2 respectively, Table 1). Sipunculids might be clustered among microbial mats as well: more than half of the sipunculids in both mosaics were distributed among microbial mats, which accounted for 38% of the mosaicked areas. The association with siboglinids was even higher, particularly in mosaic 2, where 93% of sipunculids were observed within siboglinid aggregations despite the latter constituting only 54% of the total area of the mosaic. We were unable to determine if the clustering of snails was linked to any particular associations between them and the three tested habitats of siboglinids, microbial mats and crusts or rocks (Table 1).

**Table 1:** Details on the distributions of the point marked fauna (and skate egg cases), and the three substrate/habitat types in the mosaicked active areas.

point fauna	nearest neighbor index	z-score	p-value	conclusion	No. and % on crusts	No. and % on microbial mats	No. and % on siboglinids	polygon features	area of mosaic (m^2^)	% of mosaic
**mosaic 1**										
*C. proboscidea*	0.91	−1.11	0.27	random	1 (2%)	9 (20%)	36 (82%)	crusts/rocks	11.55	3
*N. hirtipes*	0.73	−61.68	<0.001	clustered	174 (1%)	6127 (44%)	10699 (76%)	microbial mats	154.96	38
sipunculids	0.65	−48.13	<0.001	clustered	25 (0.5%)	2927 (56%)	4363 (84%)	siboglinids	282.88	70
skate eggs	0.74	−7.76	<0.001	clustered	4 (2%)	74 (30%)	199 (80%)			
snails	0.68	−8.35	<0.001	clustered	9 (5%)	61 (32%)	130 (68%)			
zoarcids	1.04	0.29	0.77	random	0 (0%)	2 (13%)	14 (88%)			
**mosaic 2**										
*C. proboscidea*	n/a	n/a	n/a	n/a	n/a	n/a	n/a	crusts/rocks	13.12	4
*N. hirtipes*	0.64	−70.60	<0.001	clustered	805 (8%)	5126 (48%)	5991 (56%)	microbial mats	115.37	38
sipunculids	0.52	−66.82	<0.001	clustered	104 (2%)	2814 (54%)	4825 (93%)	siboglinids	162.79	54
skate eggs	0.78	−2.62	0.01	clustered	1 (3%)	13 (33%)	33 (85%)			
snails	0.84	−2.34	0.02	clustered	1 (2%)	34 (55%)	45 (73%)			
zoarcids	1.14	0.59	0.56	random	0 (0%)	3 (60%)	3 (60%)			

For point features, results of average nearest neighbor analyses are listed as well as their numbers and percentages on the three substrate/habitat types. For the habitat/substrate types (crusts/rocks, microbial mats and siboglinids), total and percent areal coverage in the mosaicked areas are listed.

### Authigenic carbonate- and barite-cemented crusts

**Figure 5:**
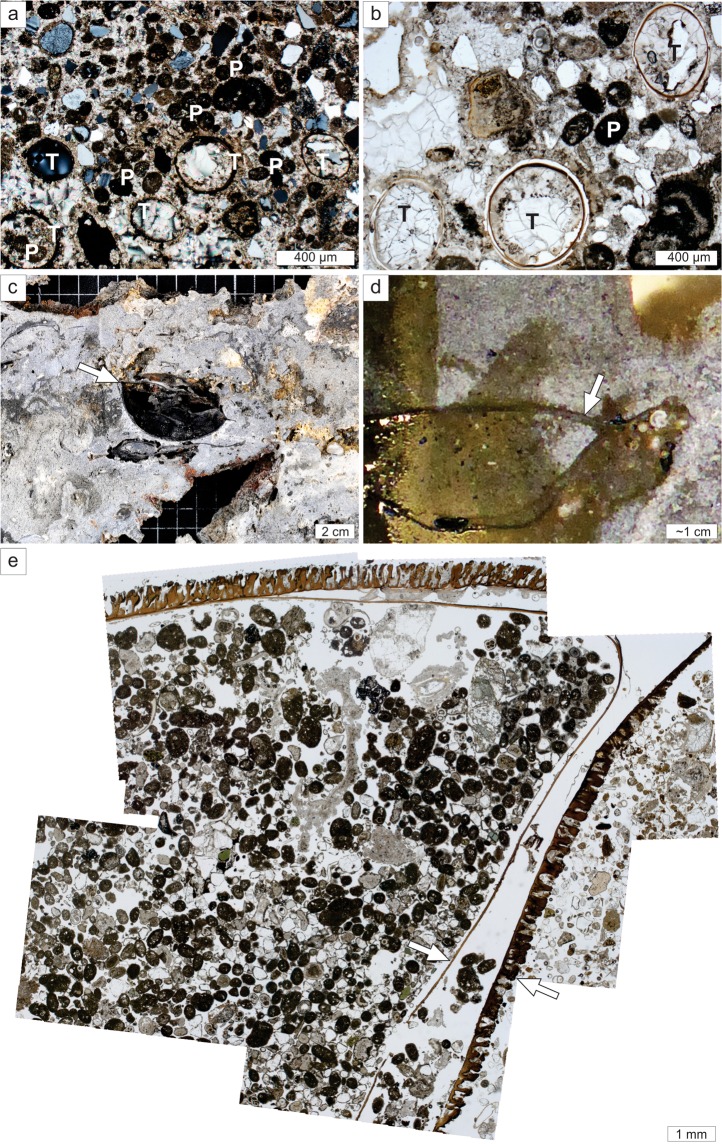
Thin-section micrographs and macroscopic images showing the textural composition of seep-carbonate crusts with cemented worms and skate eggs. (**a**) Mg-calcite cemented sediment comprising abundant grey, well-rounded to angular silt-sized quartz and feldspar grains; please note the cemented tubes (T) and abundant micritic pelloids (P); cross-polarized light (pore space appears black). (**b**) Mg-calcite cemented tubes (T), peloids (P), planktonic formanifera (upper centre), and abundant angular detrital grains; (**a**) and (**b**) sample P1710003; (**c**) Skate egg (arrow) preserved *in situ* within carbonate crust sample P1710001. (**d**) Skate egg (arrow) preserved in sample P1710003. (**e**) Micrograph mosaic showing *in situ* cemented skate egg case in (**d**); please note the double layered egg case wall (arrows); also note abundant detrital grains and micritic peloids inside the egg case.

**Figure 6:**
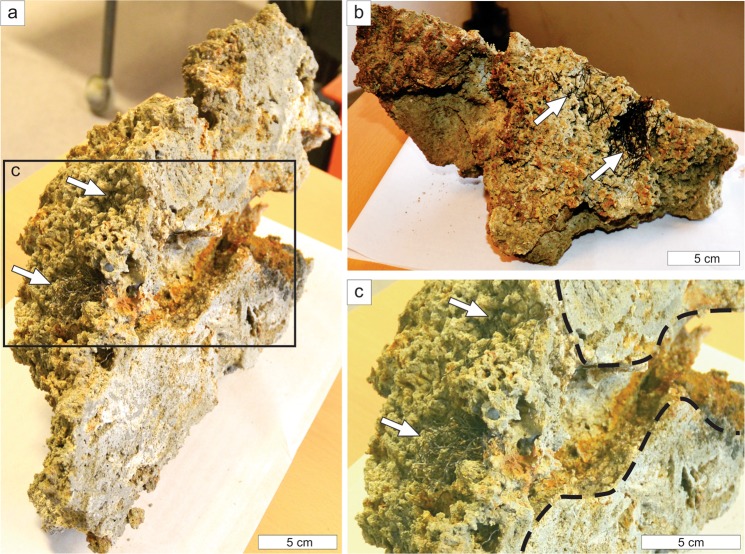
Crust sample P1710041 and associated *O. haakonmosbiensis* worms. (**a**) Oblique view of the cut surface, showing *O. haakonmosbiensis* (arrows) surrounded by cavernous seep crust; the black box depicts magnified view in (**c**). (**b**) Upper surface of the crust sample, showing anterior ends of *O. haakonmosbiensis* (arrows). (**d**) Magnified view of *O. haakonmosbiensis* bushes; please note that the buried, posterior ends of the tubes continue into a large cm–wide cavern depicted by black dashed lines.

Carbonate- and barite (BaSO_4_)-cemented crusts were present in both active and inactive areas. Numerous crust samples from active areas contained aggregations of siboglinids and the unidentified branched animal. Crusts from both inactive and active areas occasionally contained fossilized skate egg cases (Fig. 5C–E). Most crusts comprised of hemipelagic sediment, with abundant silt to sand sized quartz grains, cemented by Mg-calcite and barite. Round to ovoid and elongated micritic pelloids and faecal pellets were abundant, particularly within an *in situ* cemented skate egg case (Fig. 5). Crust δ^13^C_carb_ values were exclusively negative, ranging from −69.3 to −45.8‰ VPDB (average = −59.4 ± 6.2; N = 21, Supplementary Table S2). Similarly low δ^13^C_org_ values were measured for *O. haakonmosbiensis* tissue from individuals attached to the crusts (−60.9 to −39.1‰ VPDB, average: −52.1 ± 8.6‰ VPDB; N = 11, Supplementary Table S3). It appears that the bodies of the worms extended through the host crusts, but despite this, precipitates were absent on the tubes’ surfaces and within tube-wall laminae (Fig. 6). In particular, the worms appeared within cavernous crust portions, surrounded by well-cemented, dense sediment (Fig. 6).

### Distribution of skate egg cases and litter

Numerous skate egg cases were observed throughout the site (Fig. 4C). The cases were likely deposited by Arctic skates (*Ambylraja hyperborea*), since video footage revealed the presence of adults of this species (Fig. 4D). Within mosaic 1, 250 skate egg cases were quantified, at a density of 0.6 egg cases per m^2^. Mosaic 2 contained fewer skate egg cases: 39 in total, at a density of 0.13 per m^2^ (Fig. 3). In addition to the mosaicked areas, the video revealed a total of 287 egg cases in 8 hours and 46 minutes of footage in the two canyons (Supplementary Table S1). Of this, 5 hours and 57 minutes was footage of inactive areas and 2 hours and 49 minutes was footage of active areas. Thirty egg cases were seen in inactive areas and 257 were among active areas, indicating a preference for active areas over inactive areas as deposition locations. Within active areas, such as in the mosaics, skate egg cases appeared to display a clustered distribution, with a slight association seen with siboglinid worms (Table 1).

It should be noted that skate egg cases were often covered in sediment or half buried and therefore not clearly visible (Fig. 4C). Therefore, the number of egg cases seen in the video and images is likely an underestimation of their total abundance at the site.

A conspicuous feature of the study site is the amount of human litter. Newspapers were the most abundant, but cans, bottles and other assorted items (such as gloves) were also seen (Fig. 3, Supplementary Movie). Most articles did not appear to be particularly degraded, in fact newspaper text and brand names on bottles and cans were often perfectly legible.

## Discussion

The faunal communities at active areas of the study site are dominated by frenulate siboglinid worms and a number of heterotrophic species, but lack the large, chemosymbiotrophic animals often considered trademarks of seeps such as vestimentiferan worms, bathymodioline mussels and vesicomyid clams. This is remarkably similar to other high-latitude seep communities^10,26–31^, but a key difference is the low number of taxa (taxonomic richness) at the study site. The number of species or taxa at seeps tends to decrease with increasing water depth^4,5,32,33^. However, the seep communities of the study site are considerably poorer in taxa than deeper seep sites in the same biogeographic region for which community data is available; pockmarks on Vestnesa Ridge, NW Svalbard, and the Håkon Mosby mud volcano (HMMV), both at approximately 1200 water depth^10,27,29^. The low number of taxa at the study site in comparison to deeper seeps could represent a significant deviation from currently known and documented depth related trends of seep chemosynthesis based communities^4,33^.

A major contributor to the low richness of the active community is the absence of hard substrate dwellers, such as anemones, sponges or corals. Substrate availability is the primary limiting factor for these animals and seep carbonates are considered readily colonized substrates within the predominantly soft bottom seafloor^34–37^. Indeed, at both Vestnesa Ridge and HMMV, carbonate crusts are occupied by hard-bottom animals^10,27,29^. Abundant crust substrates suitable for colonization are present in active areas of the study site, but are notably free of cnidarians and sponges. Furthermore, these taxa are abundant on bedrock outcrops in adjacent, inactive areas (Fig. 1).

**Figure 7:**
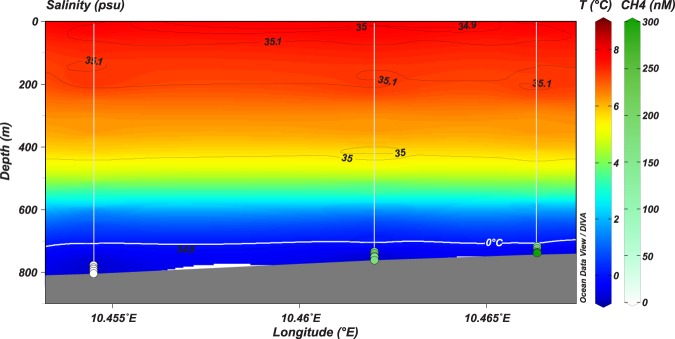
Temperature (blue to red color scale) and salinity (isolines) distribution at three stations along the transect illustrated in Fig. 1. Methane concentration is represented by the green and white colored dots at 0, 5, 10, 15 and 20 m above the seafloor.

The absence of animals colonizing the surfaces of seep crusts at the study site is therefore unexpected, and suggests a specific local environment, inhibitory to hard substrate epifauna. One possibility is a sulphidic and therefore toxic environment at the seafloor. Sulphide is characteristic of seeps, however, most is either consumed or oxidized in the sediment such that sulphide is largely undetectable in bottom water samples from seep sites^15,30,38–40^. In high flux situations, sulphide does reach the bottom water, but this tends to be localized around certain areas or discharge points^40^. We measured sulphide in all bottom water samples from the study site, with concentrations reaching up to 51 µM (Fig. 2, Supplementary Table S4). Arguably, these measurements were made among bacterial mats which tend to correspond with the high-flux locations of seeps where sulphide reaches the bottom water^40^. However, peak sulphide concentrations and the disappearance of sulphate due to reduction reactions occur shallow in the sediment which is suggestive of the zone of sulphide production (sulphate-methane transition zone, SMTZ) being located at shallow depths beneath the sediment surface (Fig. 2). The generation of sulphide so close to the sediment-water interface allows for considerable amounts of sulphide to reach the sediment surface (millimolar concentrations, Fig. 2), and subsequently, escape into the water column. The anterior portions of siboglinid tubes and the unidentified branched animal that extend a few centimetres above the sediment surface often had a white, fuzzy appearance (Fig. 4E), which in the case of the siboglinids, was confirmed through collections to be white, filamentous bacterial colonies. Since these bacteria are likely sulphide oxidizing chemoautotrophs^40–42^, their presence is consistent with sulphide in appreciable quantities above the seafloor. Sulphide was also measured in the bottom water above the core taken in an inactive region, despite only low amounts of sulphide being measured in the sediment layers of that core (Fig. 2). This could indicate the escape of sulphide into the bottom water even in low flux regions of the canyon, or considerable transport of sulphide from active areas. Additionally, methane was measured at concentrations up to 450 nM at an altitude of 5 m above the seafloor, and up to 125 nM at 25 m above the seafloor (Fig. 7, Supplementary Table S5). Since free gas bubbles are absent at the site, this is further evidence for the release of aqueous seeping fluids from the sediment to the water column (though it should be kept in mind that methane is less readily oxidized than sulphide). Together, this suggests significant concentrations of sulphide in the bottom water at the study site and particularly in active areas, which would prevent colonization of cnidarians and sponges that lack adaptations against sulphide poisoning.

Despite the absence of cnidarians and sponges, the crusts contained two taxa: siboglinid frenulate worms (*Oligobrachia haakonmosbiensis*) and the branched cnidarian or sponge or bryozoan (Fig. 4A). The former is highly unusual because frenulates are usually found on soft sediment substrates^43,44^ and in fact, this is the first record of members of this clade in association with hard substrates. Similar to other seep siboglinids such as vestimentiferans, *O. haakonmosbiensis* likely obtains the bulk of its nutrition from sulphide-oxidizing bacterial endosymbionts^45^. It is therefore possible that they release sulphate into the sediment as a waste product of symbiont driven chemoautotrophic activity, which facilitates the anaerobic oxidation of methane and therefore crust precipitation around them^46,47^. Unfortunately, we could not test this since determining whether crust precipitation is affected by siboglinid activity depends on examining the crystal forms of specifically, calcium carbonate, as opposed to the significant barite mineralization of crusts at the study site^48^.

The low δ^13^C_org_ values (−52.1 ± 8.6‰) of *O. haakonmosbiensis* associated with crusts and the similarity of these values to δ^13^C_carb_ values (−59.4 ± 6.2‰), suggests a common dependence of *O. haakonmosbiensis* and crusts from the site on strongly ^13^C-depleted carbon derived from anaerobic methane oxidation^48^. Uptake of bicarbonate ions produced by AOM processes by the worms would also prevent crust formation on the tubes of the worms themselves and indeed, the tubes of living *O. haakonmosbiensis* lacked encrustations. The occurrence of cavities within the crusts immediately surrounding the worms suggest that *O. haakonmosbiensis* releases protons as a product of sulphide oxidation which decreases local porewater pH and further prevents clogging of tubes with crust precipitates^47^. This would be necessary since encrustation would obstruct gas exchange which is hypothesized to primarily take place across the thin tube walls and epidermis of frenulates^44,49^. Therefore, crust precipitation over time encases the worms, but without blocking their transport pathways, resulting in living frenulate aggregations among the cemented crusts. The unidentified branched animal eventually colonizes the surfaces of the crusts, creating a truly unique situation whereby hard-bottom and soft-bottom animals co-occur.

Another unusual aspect of the study site is the high abundance and density of *N. hirtipes* sea spiders and their presence among microbial mats and siboglinid worm aggregations. This species is usually found on hard substrates and its diet consists of hydrozoan polyps, anemones and nudibranchs^50^, none of which were seen among them or overall, in active areas of the study site. This is the first report of *N. hirtipes* from an active seep and our results suggest that the species exhibits significant modifications to its basic life history strategies, such as habitat preference and nutrition when it is present in a seep environment. Furthermore, *N. hirtipes* at the study site is likely to be persistently exposed to elevated sulphide levels, which would require it to have mechanisms to prevent sulphide asphyxiation. Such adaptations occur in seep specialist species^51^ and has been even been suggested to be widespread among soft sediment macrofauna due to the stratification of most marine sediment into aerobic and anaerobic/reduced layers^52^. However, *N. hirtipes* is neither a seep-specialist species, nor, usually, a soft sediment dweller^50^. The possibility of it having evolved sulphide tolerance represents a paradigm shift that warrants further attention. High latitudes could provide the necessary evolutionary context. A dependence on detrital food sources in the polar deep sea would be particularly difficult during the extensive dark periods. This could have selected for heightened opportunistic tendencies and increased residence time in productivity hotspots such as seeps, which in turn could have led to the development of adaptations specific for such environments in certain species. Therefore, in high latitude regions that experience polar nights, a distinctive separation between seep-specialist and non-specialist animals, with their accompanying adaptations might be limited, which could explain why all high latitude seeps studied to date contain few (and arguably no) seep-specialist or endemic fauna^10,27,29,30^.

One of the functional features of the study site with respect to the benthic community is that the Arctic skate, *Ambylraja hyperborea* appears to be using it as an egg case nursery ground. The use of submarine canyons for this purpose has been documented for oviparous deep-water skates and canyons are hypothesized to provide optimal oceanographic conditions for skate egg case nurseries^53–57^. However, our results indicate that the canyon setting alone does not account for the observed skate egg cases: it appears that egg cases are preferentially deposited in areas of active seepage (Supplementary Table S1). To our knowledge, the use of a seep as a skate egg case nursing ground has been observed once before, at the Concepción methane seep area off Chile^58^. In this case, egg cases were mostly seen among crusts and it was hypothesized that crusts with irregular morphologies provide solid surfaces, protective nooks and crannies, and local elevation (which enhances water circulation and thereby removal of metabolic waste). However, we did not detect a particular association between egg cases and crusts at the study site (Table 1). Egg cases were also often absent from rocky surfaces and old crusts in inactive areas and together, this suggests that crusts are not the primary factor determining the use of the study site as a skate egg case nursery ground.

We therefore propose an alternative hypothesis. Though the word ‘cold’ is often used in association with seeps, they actually tend to have slightly elevated temperatures than the surrounding seafloor^4,5^. Excluding mud volcanoes, these temperature anomalies are nevertheless low, often less than 1 °C^59–61^. However, this could be significant at the study site, where water temperatures plunge to −0.7 °C (Fig. 7). Apart from the obvious risks associated with negative water temperatures, a benefit of warmer temperatures is a reduction of the lengthy incubation times of skates which could increase recruitment potential and hatching success through decreased exposure to predation^53,55^. Models show that an increase of just 0.5 °C would cut the developmental period of the Alaska skate by about 16%, or roughly 6 months^57^. Therefore small amounts of heat around seeps could have profound effects on the reproductive success of skates and in fact, deep-sea skate egg cases have been observed in association with minor increases of temperature (0.25 °C above ambient) due to diffuse flow venting around hydrothermal vents^62^.

Skate egg cases were preserved in crusts collected from the study site (Fig. 5C–E). This is indicative of the use of the study site as a nursery ground by skates over multiple generations, since carbonates are known to take hundreds to thousands of years to form^63–65^. Adults at the study site were either on the sediment or partially buried. Therefore, similar to *N. hirtipes* sea spiders, *A. hyperborea* might possess adaptations to prevent sulphide poisoning, although it should be noted that adults are capable of swimming away if conditions become lethal for them. Remarkably, this tolerance might not be restricted to the adult stage. Developing embryos in skate egg cases are exposed to external conditions on the seafloor, which, in the case of the study site would be toxic levels of sulphide. This would further support our hypothesis that benthic megafaunal species in high latitude regions have a heightened rate of exploitation of seep systems and subsequent adaptations usually seen in seep-specialist fauna in lower latitudes. Additionally it would represent a novel avenue of research with respect to the biology of *A. hyperborea* skates. In fact, a number of unique features were recorded at the study site and these unprecedented trends provide a new context for advancing our understanding of chemosynthesis-based, and polar marine ecosystem functioning and dynamics.

The functional and ecological importance of the study site should be considered with its susceptibility to human activity in mind: litter was widespread throughout the site (Fig. 3, Supplementary Movie) and the canyon walls probably function as a funnel for loose objects swept along by currents, leading to their eventual deposition and concentration within the canyons^24^. Anthropogenic waste in the oceans is common and in fact, increasing by the decade^66^. In the circum-Arctic, the density of litter has been seen to positively correlate with the level of human activity^67^. The Lofoten-Vesterålen region is a commercial hub with a large potential for seafloor litter accumulation to increase over time^68^. Decomposition in the deep sea is often slow^69,70^ and intact newspapers indicate this is true for the study site as well. Therefore, current activities alone have the potential to inundate the study site with litter, and increased human activity could hasten the process. Extensive anthropogenic waste could disrupt resident animals, and even the use of the study site as a skate egg case nursery ground. Therefore, despite its deep-water and relatively remote location, the canyon site is nonetheless highly influenced by human activity and our results reveal unanticipated ways in which it is significant for the benthos. This highlights the need for continued and persistent scientific research towards drafting effective resource management policies, particularly in the current scenario of exceptional human population growth and economic expansion.

## Methods and Materials

### Imagery and spatial analyses

Two canyons north of the Trænadjupet slide of the continental slope off Lofoten-Vesterålen were surveyed with a multibeam echosounder and HD video camera mounted to the remotely operated vehicle (ROV) Ægir 6000, aboard the R/V *G. O. Sars* (University of Bergen) in July 2017. Areas of active seepage, represented by the presence of siboglinid worms and microbial mats appeared to be patchily distributed with the canyons. Two surveys across the entire width of the south canyon were obtained. The areas visible in the video from these transects were characterized as being active or inactive (Fig. 1), to provide a rough estimate of where sites of seepage and inactive areas are located within the canyons. However, more thorough mapping is required to comprehensively understand the distribution of active and inactive areas within the two canyons.

Two assessment sites (407 and 300 m^2^) in the active areas of the southern canyon were imaged in detail to quantify abiotic and biotic features. The ROV was maintained at an altitude of 2 m above the seafloor and moved slowly, in a lawn mower fashion, to allow for overlap between successive images and between lines of images. Still images were extracted every five seconds from the downward facing video camera with the free software FFmpeg (ffmpeg.org) and time stamps were used for obtaining corresponding navigation data. Georeferenced mosaics were constructed with Agisoft’s Photoscan software (version 1.3.4 build 5067, 2017) and all visible features (animals, bacterial mats, litter and crusts or rocks) were manually marked in ArcMap 10.5. Every individual of *Nymphon hirtipes, Colossendeis proboscidea, Pandalus borealis*, ophiuroids, sipunculids, zoarcid fish, skate egg cases and litter (cans, newspapers, bottles, etc.) were marked as point features. Carbonate- and barite-cemented crusts or rocks, microbial mats and siboglinid worm aggregations were outlined (polygon features). Additionally, tiny, rissoid snails were present in large numbers among bacterial mats, and a branched animal, identifiable through the images only to the extent of being a cnidiarian, bryozoan or sponge (collections were not made) was highly abundant among siboglinid worms. The rissoid snails could only be seen if the camera was zoomed in considerably; in the mosaics, they were either not visible or appeared as pinkish smudges. The unidentified branched animal was completely intermixed among siboglinids and often blended into the background. Due the difficulties in visualizing these two taxa, they were not marked and quantified in the mosaics.

Average nearest neighbour analyses (within ArcMap 10.5) were carried out among the point fauna in order to assess their distributions (i.e., clustered, random or dispersed). Ophiuroids, zoarcids and *P. borealis* were excluded from this analysis due to their limited numbers. In this test, the null hypothesis is that the distribution in question is random and a nearest neighbour index is calculated, which is the ratio of the observed mean distance of neighbours to the expected mean distance, where the expected distance is the average distance between neighbours in a hypothetical random distribution. An index of 1 indicates a random distribution, while lower than 1 indicates clustering, and greater than 1 indicates a dispersed distribution. This test also generates p-values (probability) and z-scores (standard deviations) as measures of statistical significance with respect to the null hypothesis.

This test only indicates if a distribution is clustered, not what that clustering is associated with. Therefore, among fauna that displayed clustered distributions, location based queries were used to determine associations with different seep habitats i.e., bacterial mats, crusts/rocks and siboglinids. For each taxon, the percentage of individuals within a habitat type was compared to the percentage of the total mosaicked area covered by that habitat. For example, if siboglinids covered 20% of a mosaic, and 90% of a certain species was seen among siboglinids, it suggests an affinity of that species for siboglinids, particularly if the nearest neighbour analysis of that species indicated a clustered distribution.

Nearest neighbour analysis and location queries were also applied to skate egg cases in the mosaics. Additionally, entire dive video footage was consulted to determine whether egg cases were associated with active or inactive areas. Egg cases and adult skates were noted every time they appeared in the video and their numbers, in both active and inactive areas, were compiled and compared.

### Geochemical and CTD measurements

A total of seven push cores were taken at various locations of active seepage among microbial mats and siboglinid worm tufts within the two canyons with the ROV Ægir in 2017 (three in the north canyon and four in the south canyon, Fig. 1). Of these, one was within mosaic 2 and one was just adjacent to mosaic 1 (Fig. 3). Additionally, one core (P1710-21) was taken in an inactive area in the south canyon. This particular sampling procedure was carried out due to the limited time available during the cruise, with the aim of gaining an overall idea of geochemical conditions at the site. Sulphide (ΣHS = HS^−^ + S^2−^) and sulphate (SO_4_^2−^) concentrations were measured along the length of the cores. In four of the cores, the overlying water was also examined in order to obtain measurements on the bottom water. Methods of porewater extraction and analyses are reported in Latour *et al*.^71^. Briefly, porewater was extracted in a temperature-controlled room (4 °C) with acid-washed rhizon samplers and syringes, and 0.5 to 2 ml of porewater was preserved with Zn(OAc)_2_ onboard (<30 minutes after rhizons were disconnected) for further analyses in the lab. Samples were kept frozen all the time upon analyses. Measurements were made every 1–2 cm, with the first measurement usually having been made at the sediment-water interface. We used the ‘Cline method’ to determine sulphide concentration^72^. For most of the samples, two to four replicated measurements were performed. Uncertainty of the analysis for each sample was calculated from these replicates. The detection limit of sulphide varies between 9 and 133 μM depending on daily instrument conditions and dilution factors of the samples. We include the detection limit and the value measured for each sample in the supplementary material (Supplementary Table S4). Sulphate concentration was determined using a Dionex ICS-1100 Ion Chromatograph (IC) with a Dionex As-DV autosampler and a Dionex IonPac As23 column (eluent: 4.5 mM Na_2_CO_3_/0.8 mM NaHCO_3_, flow: 1 ml/min). The relative standard deviations from repeated measurements of different laboratory standards are better than 0.5% for concentrations above 0.1 mM. The detection limit for sulphate is ca. 0.01 mM.

Three CTD (Conductivity, Temperature, Depth) casts were performed from on board the R/V *Helmer Hanssen* in May 2018 in the south canyon using a SBE 911plus CTD. Water samples were collected 5, 10, 15, 20 and 25 m above the seafloor using Niskin bottles, and prepared for measurements of methane concentrations applying the conventional headspace gas extraction technique^73^. Immediately after recovery, water samples were transferred bubble free into 120 mL crimp seal bottles and 1 mL NaOH solution was added. Five millilitres of nitrogen were injected through the rubber septa, and the same amount of water sample was removed from the bottles. The bottles were shaken for two minutes to equilibrate the headspace nitrogen with the *in situ* water sample gas and further analysed with a Thermoscientific Trace 1310 GC equipped with a Flame Ionization Detector (FID).

### Sampling, petrography and geochemistry of crusts

Carbonate- and barite-cemented crusts were sampled from the seabed using the manipulator arms of the ROV Ægir in 2017. Dried rock samples were cut into cm–thick slabs using a wet rock saw. Thin sections (6.5 × 5 cm, 30 µm thick) were prepared from epoxy impregnated slabs and examined using standard petrographic microscopy. Samples for stable carbon isotopes were obtained from the cut slabs with a hand-held microdrill. Powders were reacted with anhydrous phosphoric acid in a GasBench II preparation line and released CO_2_ gas was analysed with a Delta V Advantage isotope ratio mass spectrometer. Crust δ^13^C values are reported in per mill (‰) relative to the Vienna Peedee belemnite (VPDB) standard. Reproducibility for δ^13^C was estimated ± 0.2‰. The mineralogical composition was determined by X-ray diffraction (XRD) on bulk-rock powders. Sample powders were analysed using a BRUKER D8 Advance diffractometer using Cu-Kα radiation at 3 to 75° 2Θ scanning angle (step size 0.02°, 1 second per step). Minerals were identified by automatic and manual peak search using the BRUKER Diffrac.EVA 3.1 software; quantification was performed applying Rietveld refinement with the TOPAS 5 software.

### Siboglinid isotope measurements

One crust sample with attached *O. haakonmosbiensis* frenulates^25^ was broken in order to retrieve the worms. All worms (11 individuals) were immediately frozen at −20 °C. In the lab, they were freeze dried in a vacuum chamber, and single individuals were separated from each other. Whole or half individual worms were packaged in tin capsules for isotope analysis. A subset were acidified with HCl and then dried overnight at 60 C before analysis to address the possibility of carbonates in the sample. Isotope values of individual dried worms were determined by continuous-flow isotope ratio mass spectrometry using a GV instruments Isoprime isotope ratio mass spectrometer (Manchester, U.K.). Precision of analyses was ± 0.1‰ based on standards used in the sample runs.

## Supplementary information

Supplementary material

Supplementary movie

## Data Availability

All data generated during and/or analyzed during this study are included in this published article (and its Supplementary Information files). Image and mosaic TIFF files and shapefiles are available from the corresponding author on request.
