# Supplementary material for: Atypical biological features of a new cold seep site on the Lofoten-Vesterålen continental margin (northern Norway)
Source: Sci Rep. 2019 Feb 11;9:1762. doi: 10.1038/s41598-018-38070-9 (PMC6370913; doi:10.1038/s41598-018-38070-9)
Supplement: Supplementary file 1 — Supplementary material [file 41598_2018_38070_MOESM1_ESM.pdf]

Atypical biological features of a new cold seep site on the Lofoten-Vesterålen continental margin (northern Norway)

Arunima Sen[1*], Tobias Himmler[1,2], Wei Li Hong[1,2], Cheshtaa Chitkara[1,3], Raymond W. Lee[4], Benedicte Ferré[1], Aivo Lepland[1,2] and Jochen Knies[1,2]

[1]Centre for Arctic Gas Hydrate, Environment and Climate (CAGE), Department of Geosciences, UiT-The Arctic University of Norway in Tromsø, Tromsø, Norway

[2]Geological Survey of Norway (NGU), Trondheim, Norway

[3]Faculty of Science and Technology, University of Basque Country, Leioa-Bilbao, Spain

[4]School of Biological Sciences, Washington State University, Pullman, WA, U.S.A.

*** Correspondence:**

Arunima Sen

arunima.sen@uit.no

Supplementary Table S1: Numbers of adult *Ambylraja hyperborea* skates and egg cases seen in the video, separated by their presence in areas of active seepage and non-seep locations.

video #	time in inactive areas (hh:mm:ss)	number of adult skates	number of skate egg cases	time in active areas (hh:mm:ss)	number of adult skates	number of skate egg cases
1	00:27:40	0	2	00:32:20	1	24
2	00:30:26	2	13	00:29:22	2	60
3	00:31:14	1	4	00:20:36	0	30
4	00:18:21	0	5	00:10:28	0	27
5	00:00:00	0	0	00:10:48	0	12
6	00:00:00	0	0	00:02:27	0	2
7	00:21:25	0	4	00:11:27	0	19
8	00:18:13	0	1	00:12:25	0	17
9	00:04:43	0	0	00:03:39	0	2
10	00:22:06	0	1	00:06:23	0	2
11	00:00:00	0	0	00:00:23	0	1
12	01:00:00	0	0	00:19:45	0	35
13	02:00:00	0	0	00:00:39	0	3
14	00:02:55	0	0	00:08:37	0	23
**Total**	**05:57:03**	**3**	**30**	**02:49:19**	**3**	**257**

Supplementary Table S2: Detailed locations and stable carbon isotopic composition of crust samples sampled from the seabed.

**Sample**	**Canyon**	**Latitude (N)**	**Longitude (E)**	**Water depth (m)**	**Microfacies**	**$\delta^{18}C$ (‰ VPDB)**
P1710001-A	south	68.1585	10.4609	795	fibrous cement	−69.3
P1710004-1	south	68.1586	10.461	797	fibrous cement	−65.4
P1710005-3	south	68.1583	10.4608	792	fibrous cement	−65.5
P1710023-3	south	68.1584	10.4608	795	fibrous cement	−60.4
P1710026-2	south	68.1584	10.4602	583	fibrous cement	−59.1
P1710041-1	south	68.1583	10.4606	795	fibrous cement	−60.2
P1710001-A	south	68.1585	10.4609	795	matrix	−59.7
P1710004-1	south	68.1586	10.461	797	matrix	−57.7
P1710005-3	south	68.1583	10.4608	792	matrix	−66.3
P1710006-1	south	68.1583	10.4606	795	matrix	−65.9
P1710023-3	south	68.1588	10.456	799	matrix	−56.9
P1710025-1	south	68.1589	10.4564	801	matrix	−57.9
P1710026-2	south	68.1589	10.4561	801	matrix	−54.1
P1710037-1	south	68.1578	10.4654	769	matrix	−67.3
P1710037-1	south	68.1578	10.4654	769	matrix	−60.4
P1710041-1	south	68.158	10.4658	777	matrix	−55.7
P1710042-1	south	68.158	10.4659	777	matrix	−61.8
P1710044-2	north	68.1667	10.4646	805	matrix	−45.8
P1710044-2	north	68.1667	10.4646	805	matrix	−46.4
P1710046-2	north	68.1669	10.4645	804	matrix	−54.8
P1710047-1	north	68.1668	10.4641	806	matrix	−57.4

Supplementary Table S3: Stable carbon isotope measurements of *O. haakonmosbiensis* individuals from a crust.

sample	acid treatment	$\delta^{13}C$ ‰ VPDB
1	acidified	-39.14
2	acidified	-40.30
3	not acidified	-38.43
4	acidified	-55.58
5	acidified	-58.71
6	acidified	-58.22
7	acidified	-60.92
8	not acidified	-52.67
9	not acidified	-59.35
10	not acidified	-55.33
11	not acidified	-54.29

Supplementary Table S4: Complete geochemical data (sulphide and sulphate concentrations) from the 8 push cores taken at the study site. BD = below detection limit, NA = not available. 0 in the depth column represents the sediment-water interface.

core	Depth	ΣHS (mM)	ΣHS (µM)	SO_4^{2-} (mM)	std. dev. (µM)	detection limit (µM)
P1710-9	0.5	2.2	2168	9.9	26	21
	2	2.6	2616	0.8	149	21
	3	3.0	3034	0.4	76	21
	4	2.6	2604	0.6	144	21
	5	3.0	3028	0.3	298	21
	6	3.5	3512	0.2	239	35
	7	2.7	2702	0.6	73	21
	9	2.7	2741	0.9	96	21
	11	3.6	3614	0.9	26	35
	13	2.2	2193	1.0	81	35
	19	1.6	1573	0.5	13	35
P1710-12	0	1.2	1183	24.1	NA	45
	2.5	2.0	2028	21.2	NA	45
	5.5	2.9	2921	6.6	NA	76
	8.5	0.2	225	2.2	NA	85
	10.5	0.4	368	0.2	NA	85
	13	0.0	24	0.5	NA	15
	15.5	0.0	39	NA	64	22
	25	0.0	43	BD	42	22
	30	0.0	18	0.2	47	15
P1710-16	bottom water	0.0	13	28.4	NA	9
	2	3.1	3089	19.1	NA	45
	4	4.1	4116	13.5	NA	45
	6	5.3	5300	3.7	195	45
	8	5.0	5043	0.1	923	45
	12	5.0	4957	0.0	306	45
	16	4.2	4229	BD	217	45
	20	3.2	3168	BD	202	45
	24	4.7	4653	0.1	372	45
	28	4.9	4905	0.1	256	45
P1710-21	bottom water	0.0	11	27.3	NA	9
	0.5	0.0	12	28.1	NA	9
	2.5	0.0	12	28.5	NA	9
	4.5	0.0	13	27.7	NA	9
	6.5	0.0	11	28.2	NA	9
	8.5	0.0	11	28.2	NA	9
	12.5	0.0	13	28.2	NA	9
	16.5	0.0	11	27.7	NA	9
	20.5	0.0	11	27.7	NA	9

	24.5	0.0	13	27.8	NA	9
	26.5	0.0	11	27.2	NA	9
P1710-30	bottom water	0.1	51	28.0	NA	16
	0	0.8	780	19.4	140	80
	1	1.5	1469	13.4	588	63
	2	1.6	1562	8.6	720	95
	3	2.4	2373	5.3	134	80
	4	2.2	2232	4.9	136	80
	5	2.5	2457	2.1	172	133
	6	2.4	2407	0.9	360	133
	8	2.0	1955	0.6	96	133
	12	2.0	1963	0.2	100	133
	14	2.5	2541	BD	173	80
	16	2.4	2447	0.2	229	133
	21	1.1	1097	1.5	242	133
	24	1.4	1360	BD	97	80
	26	0.6	649	BD	39	133
	28	0.1	68	BD	37	48
	30	0.1	68	BD	45	16
	34	0.1	92	BD	91	27
	37	0.1	93	0.1	83	27
P1710-52	1	0.4	358	24.9	47	80
	5	0.9	942	22.7	480	133
	9	0.9	947	21.0	313	133
	13	0.1	114	1.3	94	27
	15	0.1	120	2.5	109	41
	19	0.3	282	0.5	322	41
	21	0.2	214	NA	213	59
	23	0.2	192	0.4	62	27
	25	0.3	291	0.4	280	77
	27	0.3	324	0.4	5	27
	31	0.2	162	NA	102	32
	37	0.3	324	7.3	44	27
P1710-56	0	0.8	839	14.4	175	35
	1	0.7	681	14.6	344	70
	2	NA	NA	15.8	NA	NA
	3	1.4	1431	1.7	315	54
	4	0.8	783	NA	125	54
	5	1.1	1076	6.9	349	66
	7	0.3	318	7.6	70	35
	10	0.4	354	4.6	11	35
	12	0.0	24	0.2	65	35
	14	0.0	18	NA	NA	21
	16	0.0	31	0.2	NA	35
	18	0.0	25	NA	NA	35
	20	0.0	13	0.1	NA	78

	22	0.0	33	BD	NA	35
	24	0.0	35	BD	NA	35
	29	0.0	26	BD	NA	35
	30	0.0	42	0.1	NA	35
	9	0.5	457	11.9	NA	35
P1710-57	bottom water	0.0	7	28.5	NA	7
	2	2.1	2056	10.7	650	76
	4	2.9	2853	5.6	1015	76
	7	2.5	2498	7.4	700	35
	9	1.8	1775	8.6	28	35

Supplementary Table S5: Methane concentrations of water samples taken with Niskin bottles.

Station (CTD cast)	Bottle	Longitude	Latitude	Temperature (˚C)	Depth (m)	Altitude (m)	CH_4 (nM)
791 (west cast)	1	10.46229	68.15843	-0.733	757	0	178
791 (west cast)	2	10.46229	68.15843	-0.7357	750	5	149
791 (west cast)	3	10.46229	68.15843	-0.7327	747	10	220
791 (west cast)	4	10.46229	68.15843	-0.7305	742	15	200
791 (west cast)	5	10.46229	68.15843	-0.6771	737	20	159
792 (central cast)	1	10.4664	68.15834	-0.6494	739	0	448
792 (central cast)	2	10.4664	68.15834	-0.634	735	5	432
792 (central cast)	3	10.4664	68.15834	-0.5032	730	10	54
792 (central cast)	4	10.4664	68.15834	-0.4746	724	15	191
792 (central cast)	5	10.4664	68.15834	-0.3899	720	20	125
793 (west cast)	1	10.45449	68.15866	-0.7604	800	0	2
793 (west cast)	2	10.45449	68.15866	-0.76	794	5	2
793 (west cast)	3	10.45449	68.15866	-0.7598	790	10	1
793 (west cast)	4	10.45449	68.15866	-0.7594	786	15	2
793 (west cast)	5	10.45449	68.15866	-0.736	780	20	1

Supplementary movie: Sample video of an active area of the study site where the chemosynthesis based community, skate egg cases and items of litter are visible.
